# Supplementary material for: Structural Assembly of Qβ Virion and Its Diverse Forms of Virus-like Particles
Source: Viruses. 2022 Jan 24;14(2):225. doi: 10.3390/v14020225 (PMC8880383; doi:10.3390/v14020225)
Supplement: Supplementary file 1 [file viruses-14-00225-s001.zip › viruses-1562721-supplementary-revised/viruses-1562721-Supplementary_information-revised.pdf]

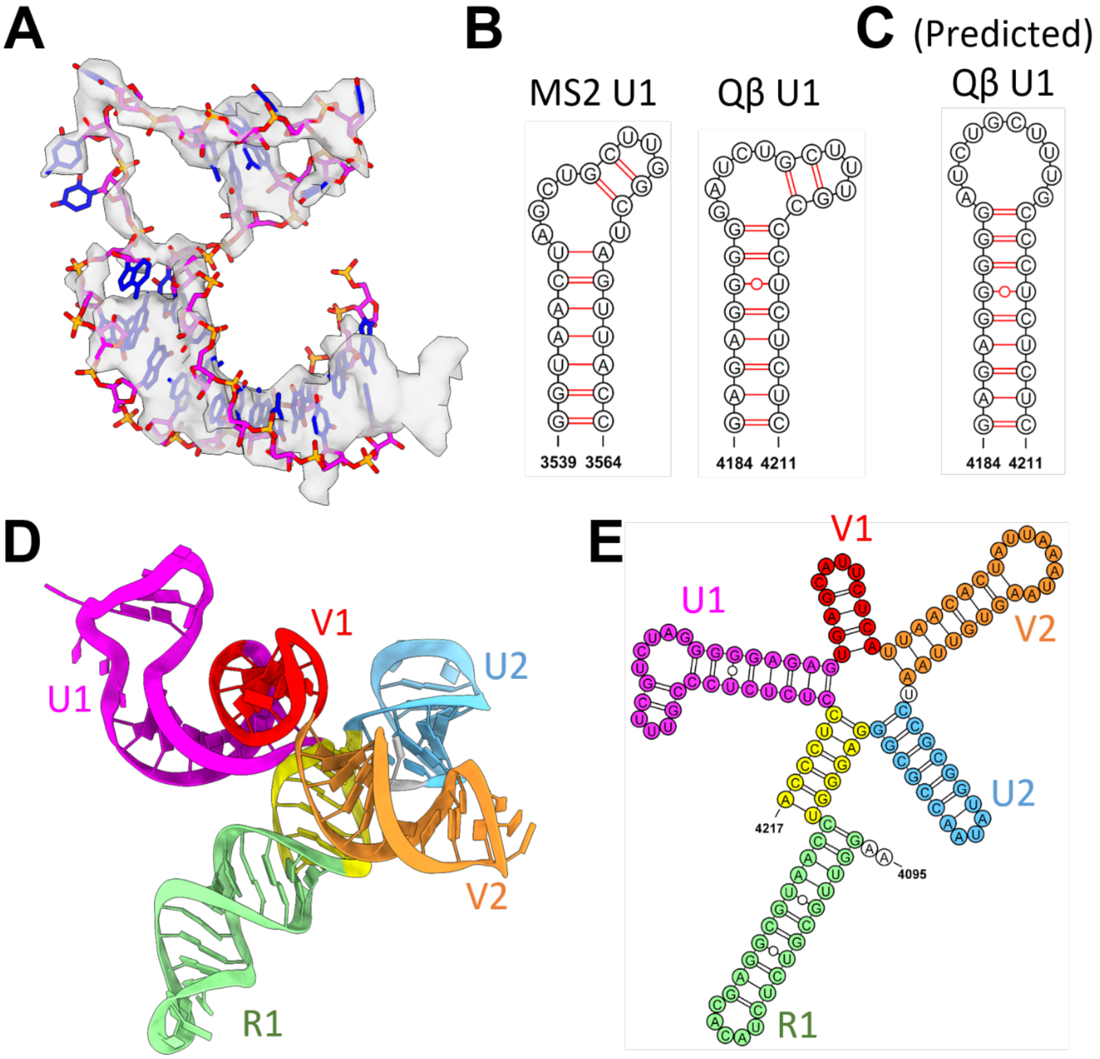


**Figure S1.** The 3′ UTR of the Qβ gRNA. (A) The model of the U1 in the Qβ 3′ UTR with its cryo-EM density. (B) The secondary structures of the U1 in the MS2 and Qβ 3′ UTR observed in the experimental data. (C) The theoretically predicted secondary structure of the U1 in the Qβ 3′ UTR. (D) The model of the 3′ UTR in the Qβ. The sequences of R1, U2, V2, U1, and the connecting helix are colored green, blue, orange, red, magenta, and yellow, respectively. (E) The secondary structure of the 3′ UTR in Qβ. Each helical stem is colored as in Panel D.

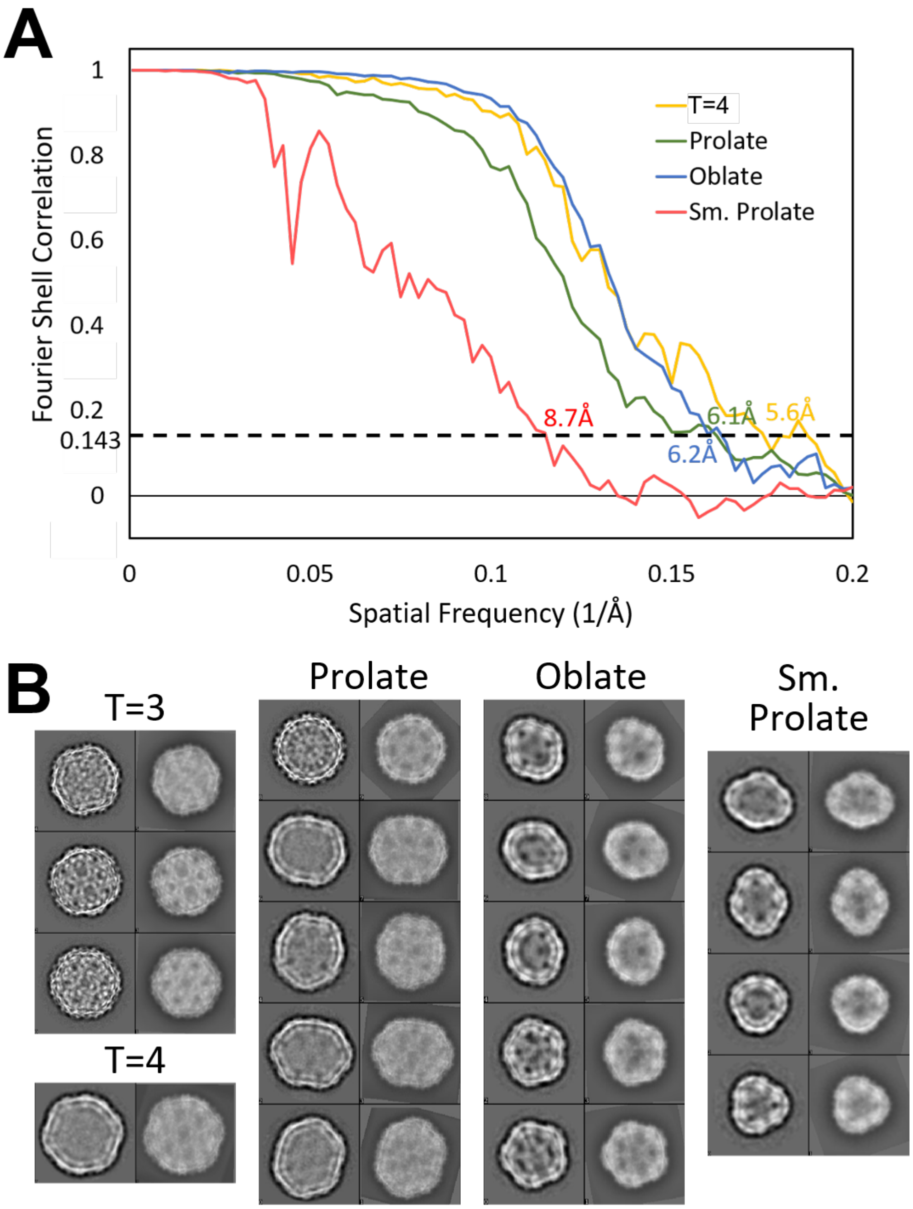


**Figure S2.** The Fourier Shell Correlation (FSC) curves and the 2D class averages of Qβ VLPs. (A) The FSC curves for the single-particle cryo-EM maps of the Qβ VLP with *T*=4, prolate, oblate, and small prolate conformations are shown in yellow, green, blue, and red, respectively. (B) Refinement results against class averages of each VLP form. The left column of each form shows the projections of the refined map with different orientations. The right column shows the 2D class averages matching the projections in the left column.

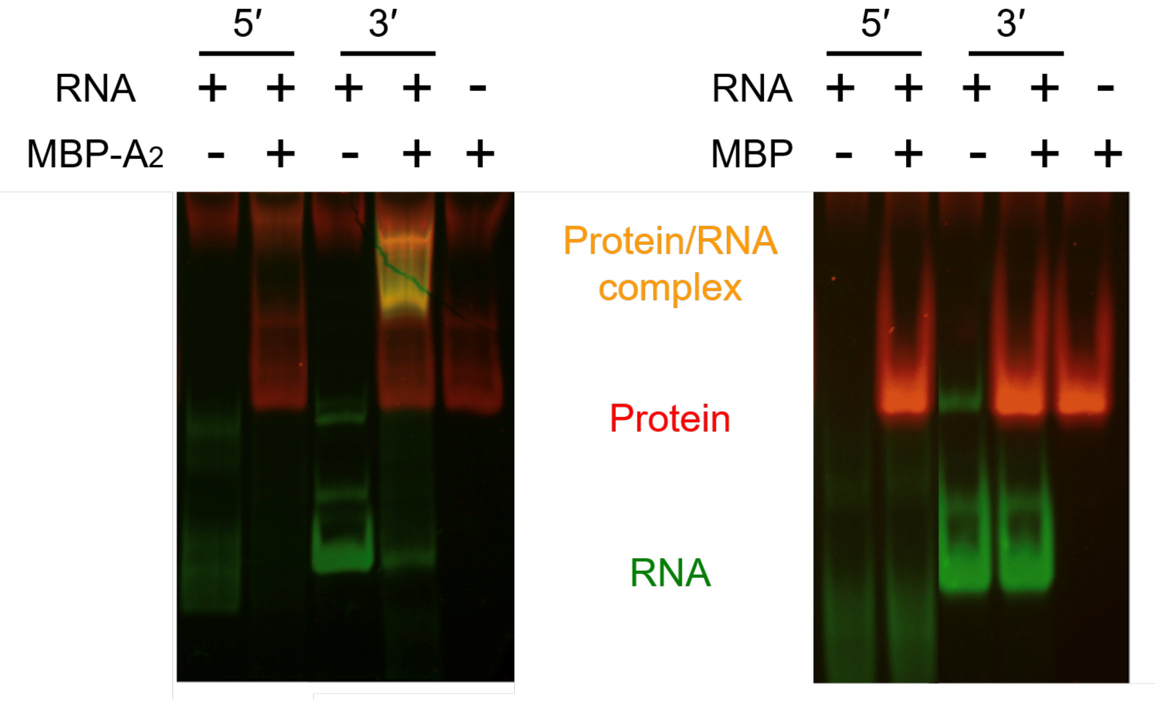


**Figure S3.** The Mat ($A_2$) interacts with the 3′ UTR of Qβ. (A) Electrophoretic Mobility Shift Assay (EMSA) of the 5′ and 3′ UTR with the Maltose-binding protein (MBP) tagged $A_2$. The gRNA fragments of the 5′ UTR (residues 1-60) and the 3′ UTR (residues 4126-4217) were stained with SYBR™ Green (green). The protein was stained with SYPRO™ Ruby (red). The co-localized band (yellow) of the RNA and protein at the lane of 3′ represents the interaction of the 3′ UTR and the Mat. (B) As a control, EMSA of the 5′ and 3′ UTR with the MBP tag shows no interaction between the RNA and the MBP.

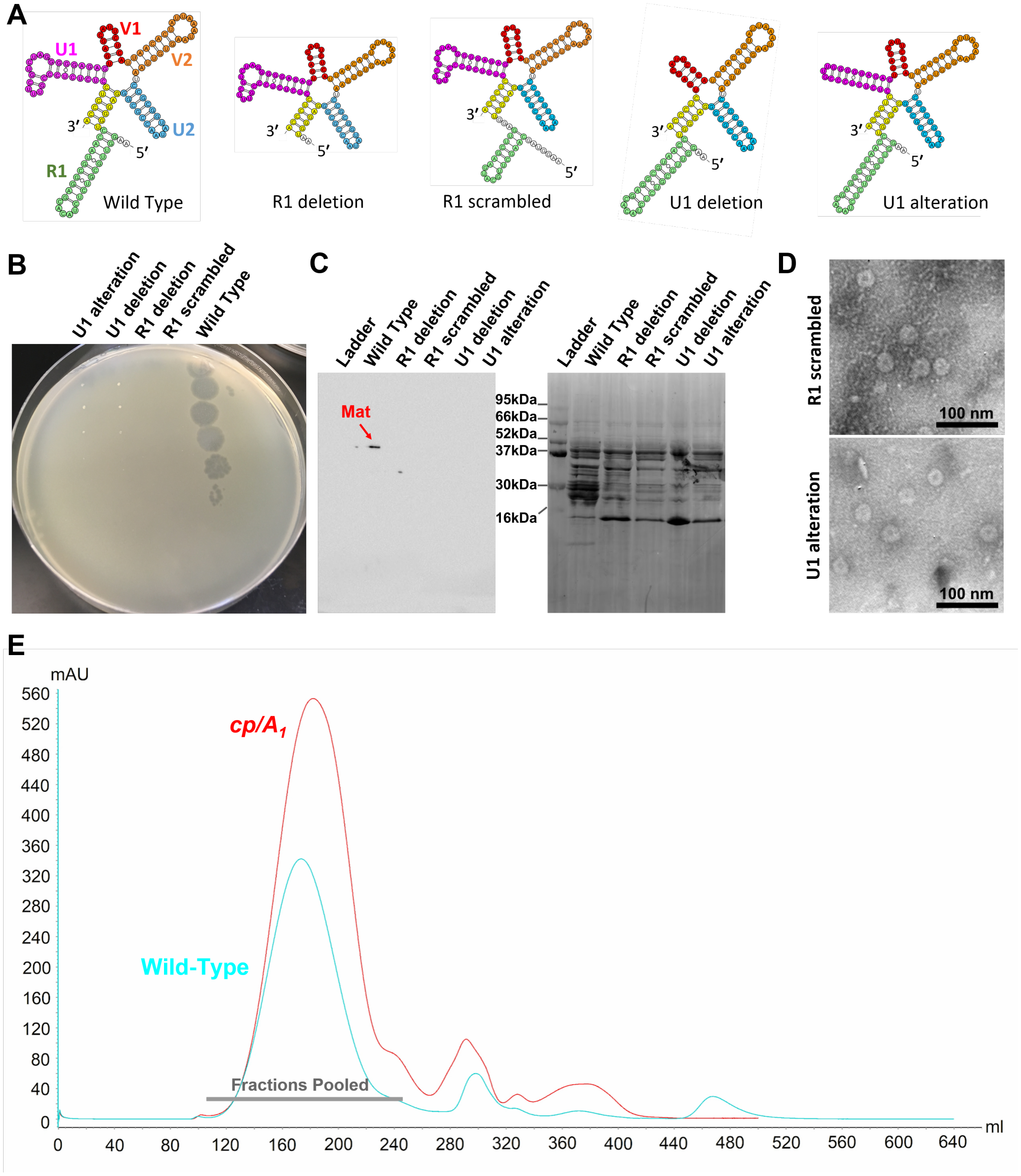


**Figure S4.** Specific RNA stem-loops at the 3′ end of the gRNA are required for the formation of infectious particles; and the purification of ssRNA phages and VLPs using gel-filtration chromatography. (A) Based on the 3′ end of the wild-type gRNA, four mutants were engineered on a cDNA plasmid to validate the model of the gRNA. The R1 loop, which binds the internal CP dimer, was either deleted (R1 deletion, second from left in Panel A) or re-engineered to maintain coding capacity but with an alternative RNA structure (R1 scrambled, middle in Panel A). For U1, which does not code for any proteins and binds to the Mat, two mutations were engineered, such that the entire stem-loop was deleted or the loop was altered (U1 deletion and U1 alteration, second from the right and on the right, respectively). (B) Titers from overnight cultures of bacteria show that the mutants are all non-infectious, while *E. coli* with the wild-type plasmid produces infectious particles. (C) A Western Blot using an antibody against Mat ($A_2$) shows that CsCl purified

particles produced from these mutants do not incorporate the $A_2$. Wild-type and mutants were produced from basal level expression off of a plasmid in female *E. coli* (F-), to prevent normal infectious replication. The left panel is the Western Blot of the mutants and wild-type, while the right panel is the same membrane, stained after the blot to show total protein. Basal level expression produces low amounts of phage with host proteins seen in the stained membrane. (D) Two of the mutants, R1 scrambled and U1 alternation, were purified and the negative-stain EM showed that particles were formed. (E) Wild-type produced phage from an infection (cyan) or the *cp/A*$_1$ overexpressed (red) purified over an S500 column which was used for subsequent structural analyses.

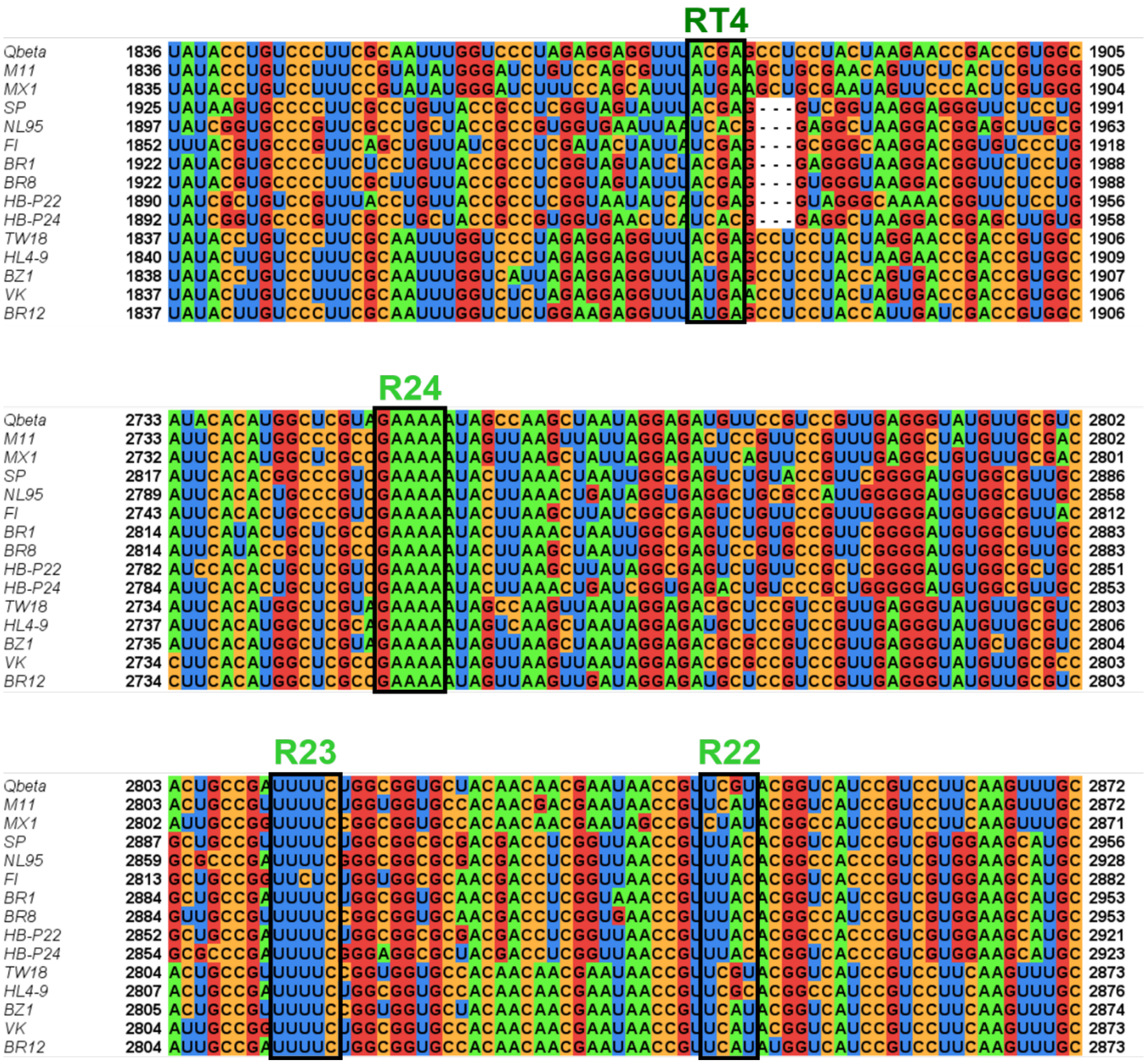


**Figure S5.** Conservation of the two kissing-loops in the Qβ-like phages. R22, R23, and R24 are at the replicase gene. RT4 is at the read-through gene.

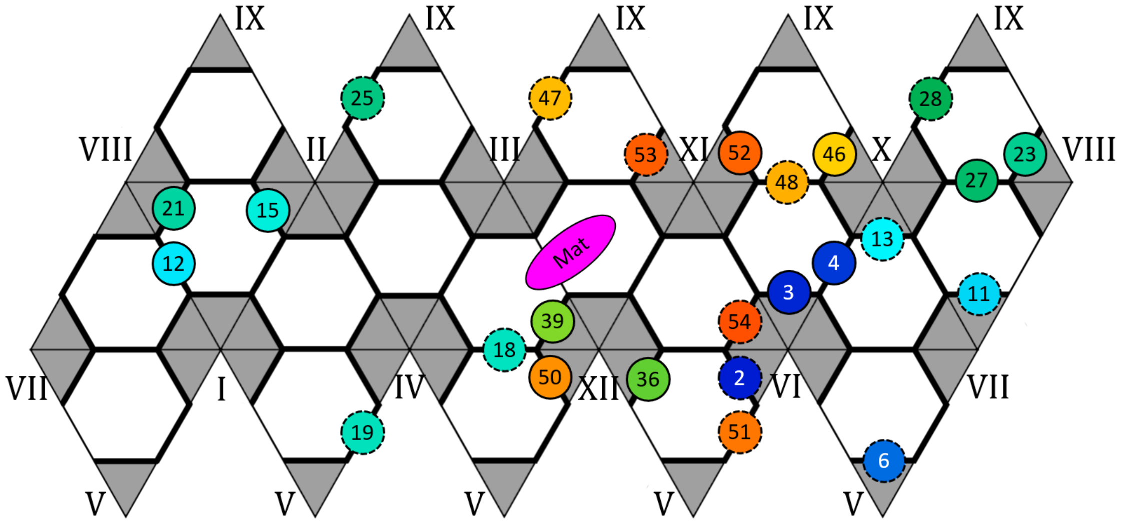


**Figure S6.** CP-sandwiched and CP-anchored RNA stem-loops. Similar to Figure 2B, the RNA stem-loops are labeled with circles on the unwrapped capsid with each thick line representing a coat protein dimer. The solid and dash circles represent the CP-sandwiched and CP-anchored stem-loops, respectively (Table S2 and S3). The circles are colored rainbow by their related sequence as Figure 2D from the 5′ to 3′ ends.

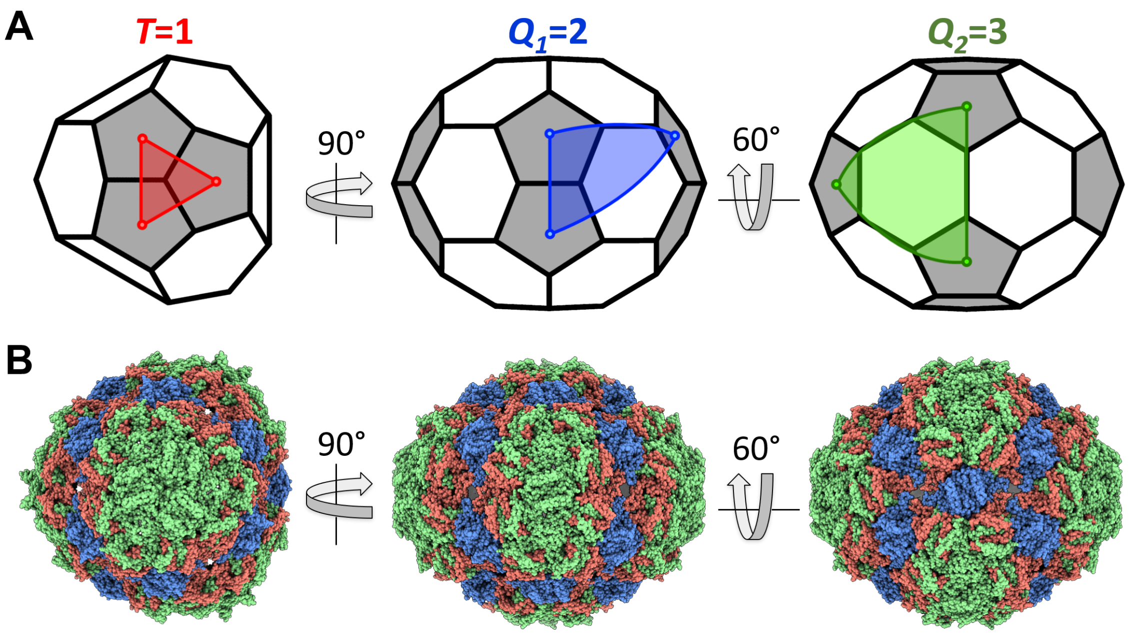


**Figure S7.** The triangulation numbers of the small prolate VLP. (A) The cage showing the triangulation number of each face. Pentamers are colored grey. The facet triangles of $T$=1, $Q_1$=2, and $Q_2$=3 are colored red, blue, and green, respectively. (B) Models of the small-prolate VLP viewing from the orientations corresponding to the orientations of the cages in Panel A.

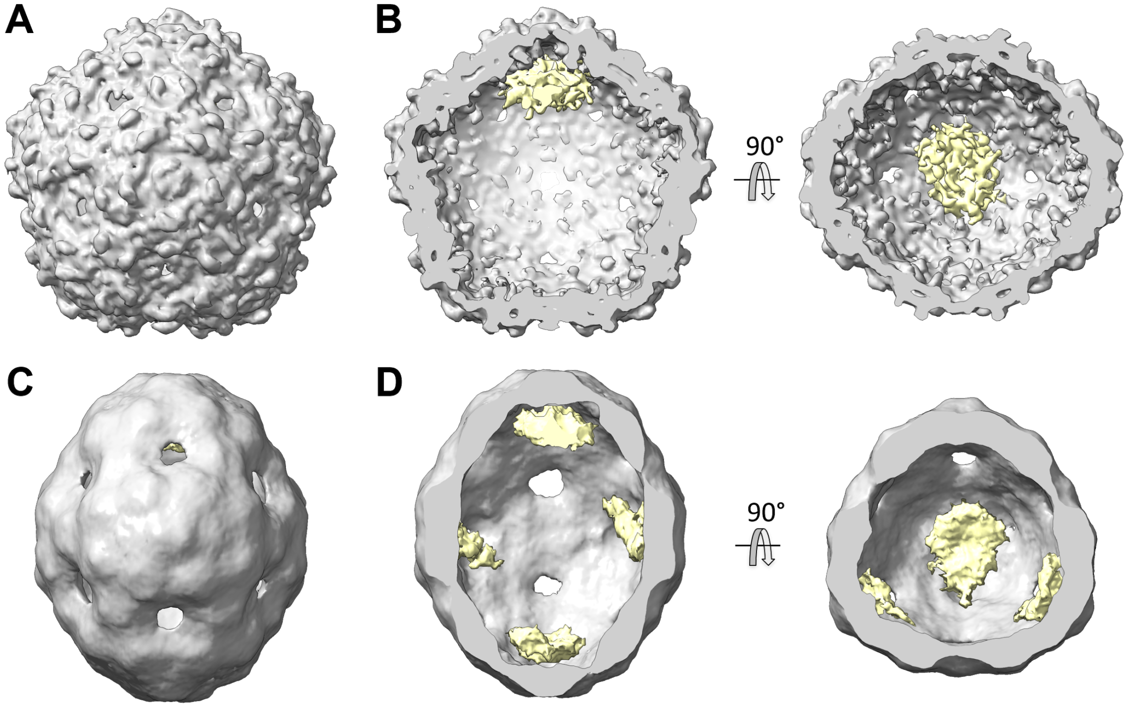


**Figure S8.** The extra densities inside the oblate and small prolate VLPs. (A) An asymmetric cryo-EM reconstruction of the oblate VLP. (B) Cut-through views of the asymmetric oblate map. (C) An asymmetric cryo-EM reconstruction of the small prolate VLP. (D) Cut-through views of the asymmetric small prolate map. The density of the capsid is color grey. The extra density inside the capsid is colored yellow.

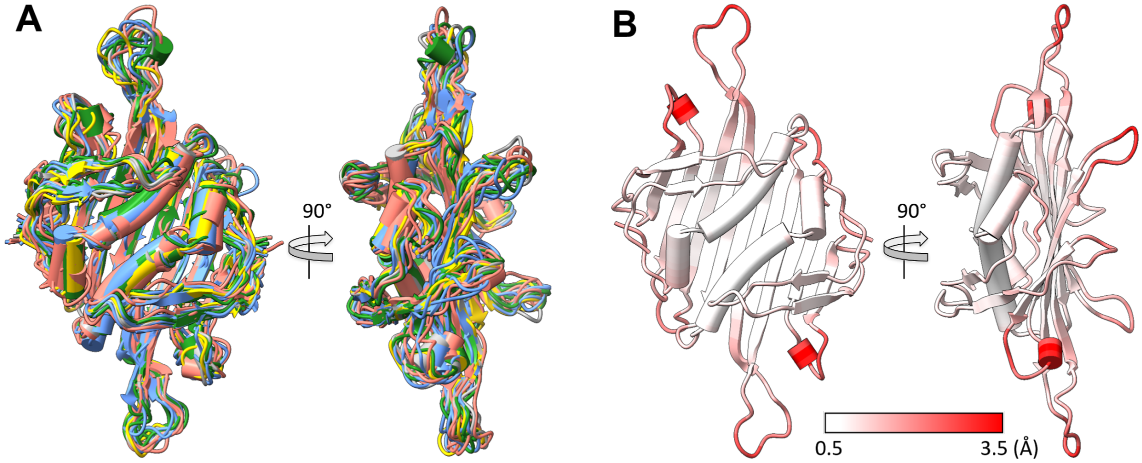


**Figure S9.** The comparison of CP dimers in different forms of Qβ VLPs. (A) Aligned CP dimers from different forms of Qβ VLPs. The CP dimers in the forms of *T*=3, *T*=4, prolate, oblate, and small prolate are colored grey, yellow, green, blue, and red, respectively. (B) The flexibility of CP dimers. The color scale, from white to red, represents the root-mean-square deviation of C$\alpha$ positions for each residue among CP dimers extracted from different forms of VLPs.

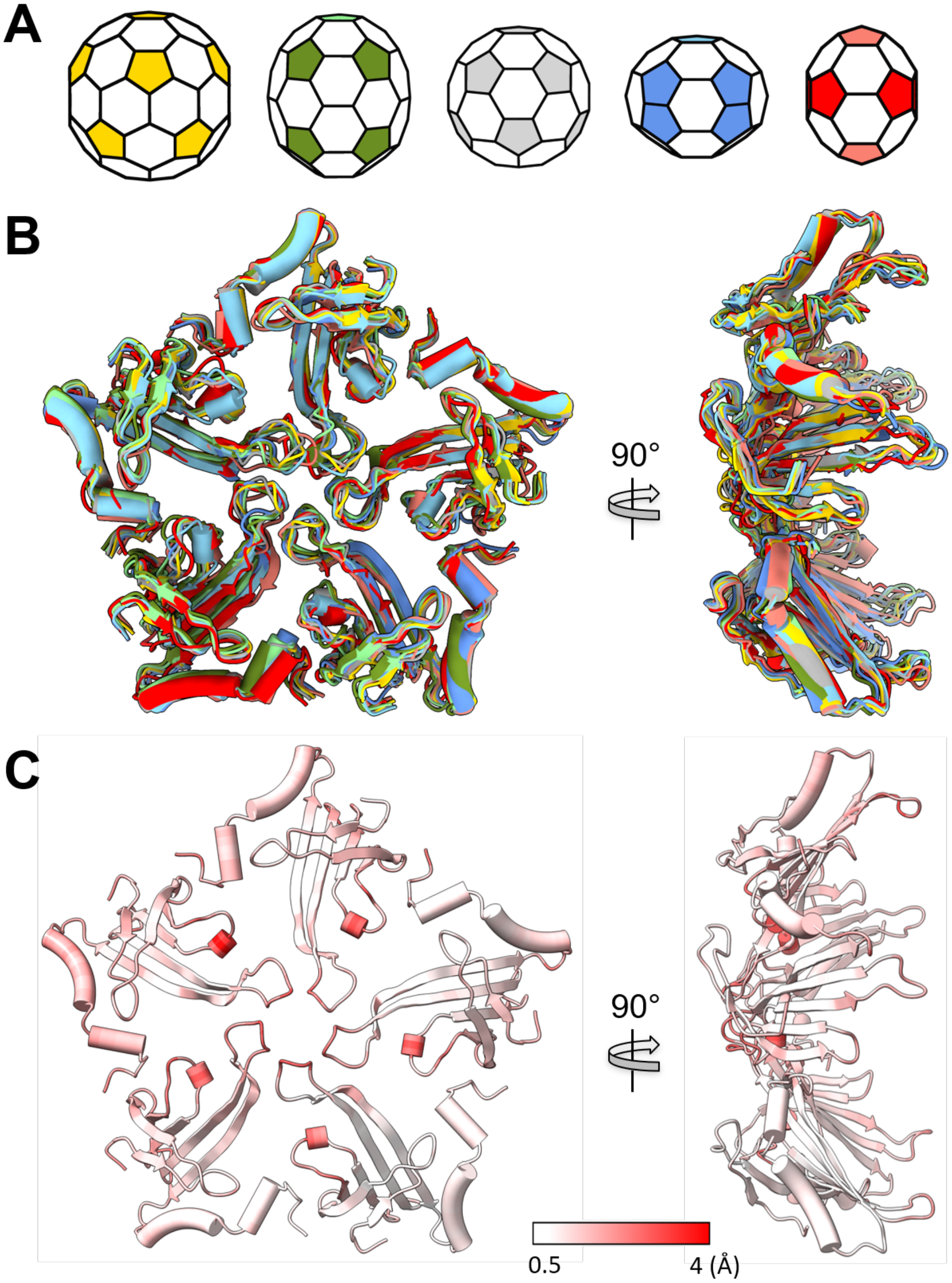


**Figure S10.** The comparison of pentamers in different forms of Qβ VLPs. (A) The cages of different forms of VLPs with the pentamers colored. (B) Structure overlay of the pentamers in each form against the pentamer in the *T*=3 VLP. Each pentamer is colored by its corresponding color in Panel A. (C) The flexibility of pentamers. The color scale, from white to red, represents the root-mean-square deviation of C$\alpha$ positions for each residue among pentamers extracted from different forms of VLPs.

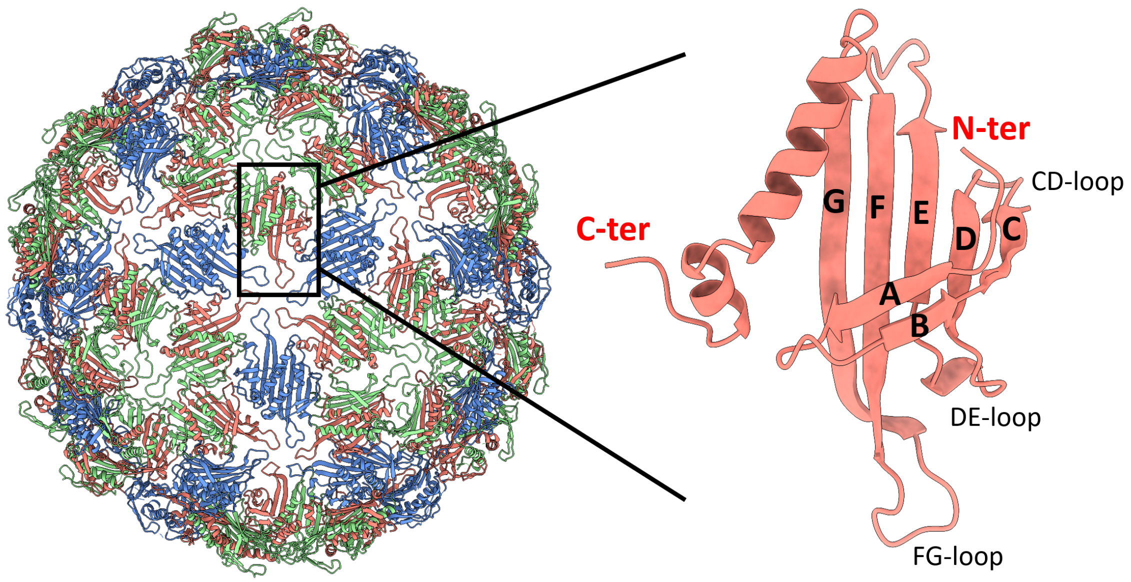


**Figure S11.** The structure of the Qβ CP monomer. A Qβ CP monomer (right) is extracted from the capsid (left). The β-strands are labeled from A to G. The CD-loop, DE-loop, FG-loop, N- and C-termini are also labeled.

**Table S1.** Operator-like RNA stem-loops. (A) To compare the geometry of these stem-loops with the actual operator relative to the CP-dimers, we aligned the CP-dimers of these stem-loop-CP-dimer complexes to the operator-CP-dimer complex (Number 34) and Gaussian-blur the models of the RNA fragments into densities at the 6-Å resolution, with which we can calculate a cross-correlation score. The operator-like stem-loops have cross-correlation scores larger than 0.55 to the density of the operator. Number 34 is the operator stem-loop. Number 57 is the stem-loop interacting with the internal CP dimer. Each stem-loop-CP-dimer complex is shown in two orthogonal views (left and right, respectively). The residue numbers for each stem-loop are indicated in the parenthesis. (B) Secondary structures and sequence motifs of operator-like RNA stem-loops are more diverse than the previously proposed operator sequence features for binding Qβ CP dimer[1].

**A**

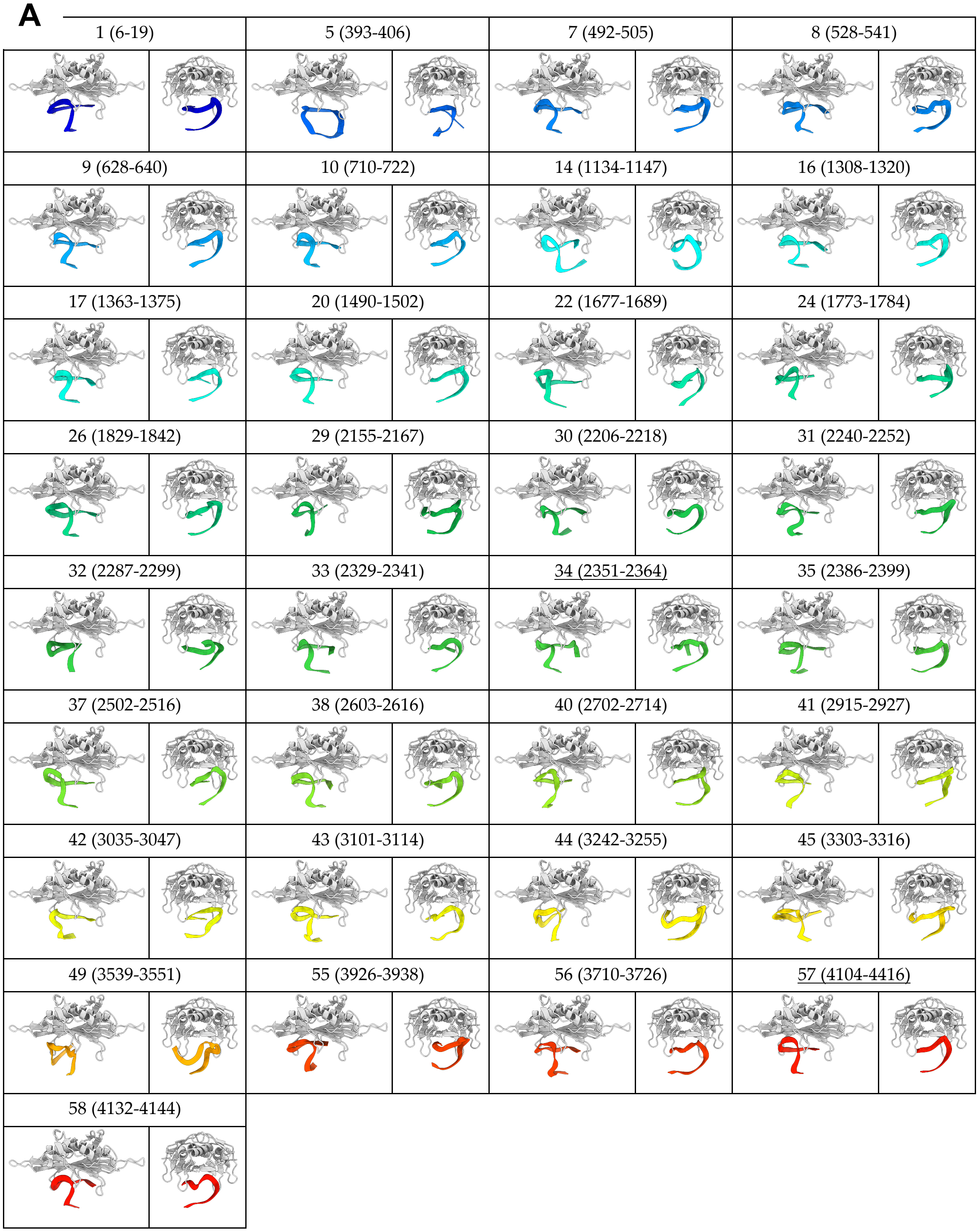

**B**

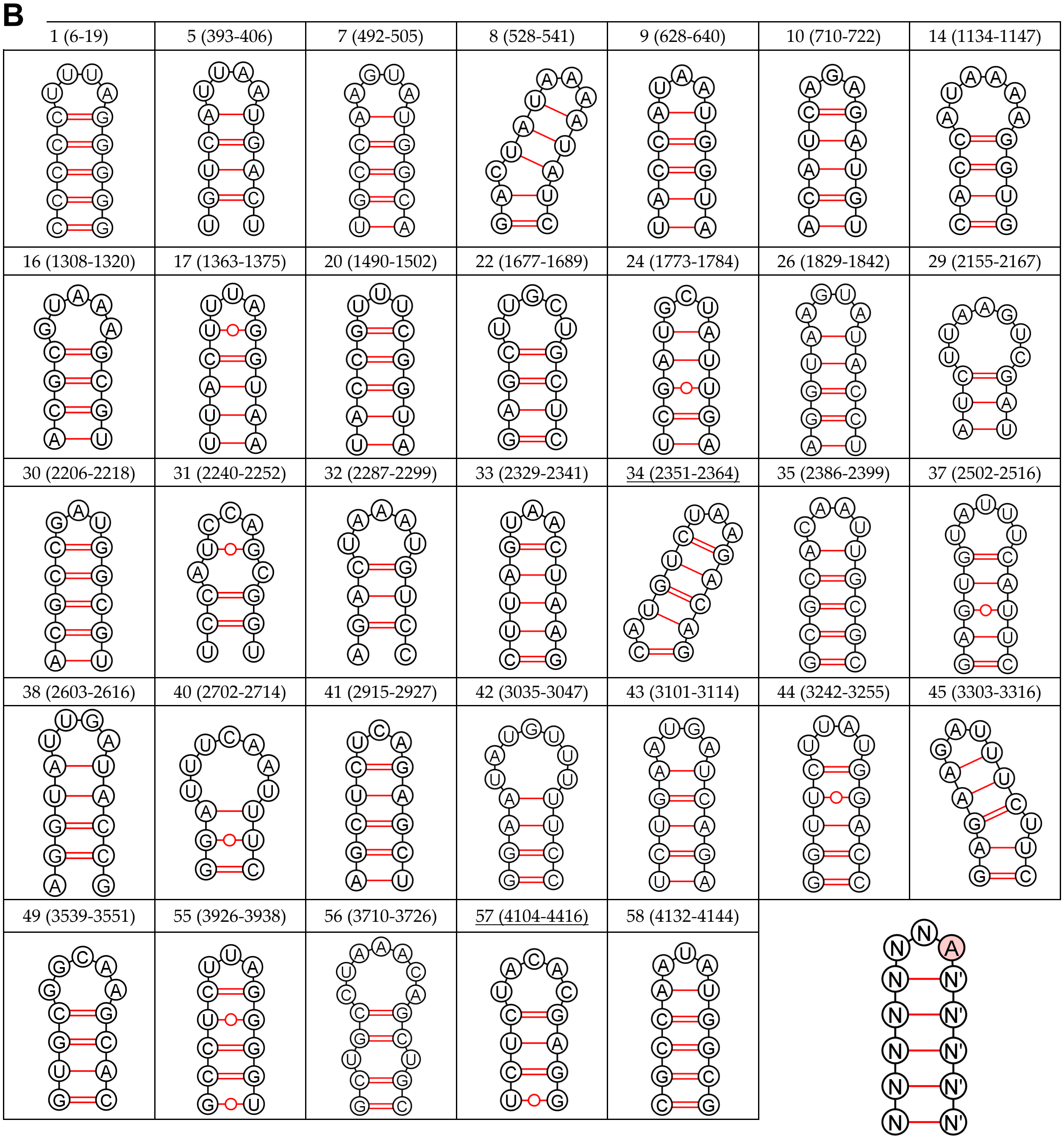


proposed features for operator-CP binding [1]

**Table S2.** CP-sandwiched RNA stem-loops. They have cross-correlation scores between 0.30 and 0.55 to the density of the operator. Each stem-loop-CP-dimer complex is shown in two orthogonal views (left and right, respectively). The residue numbers for each stem-loop are indicated in the parenthesis.

| 3 (128-140) | 4 (220-232) | 12 (937-949) | 15 (1267-1280) |
|---|---|---|---|
| 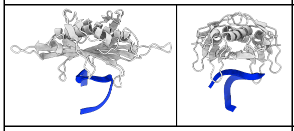 | 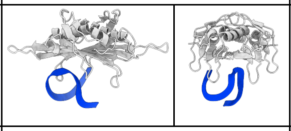 | 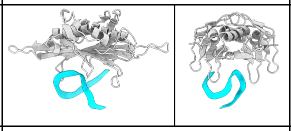 | 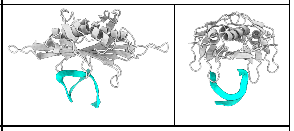 |
| 21 (1622-1634) | 23 (1711-1723) | 27 (1954-1966) | 36 (2503-2515) |
| 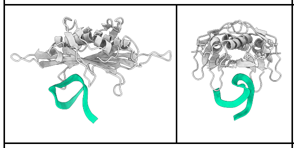 | 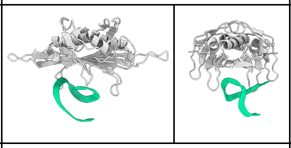 | 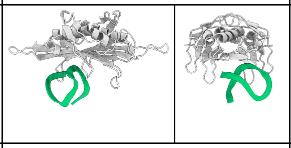 | 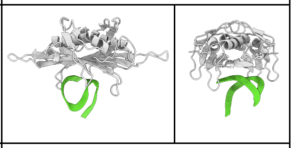 |
| 39 (2629-2641) | 46 (3349-3362) | 50 (3623-3636) | 52 (3794-3807) |
| 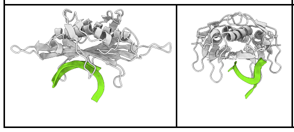 | 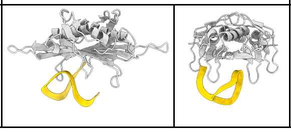 | 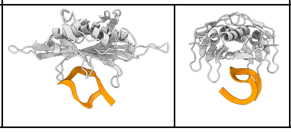 | 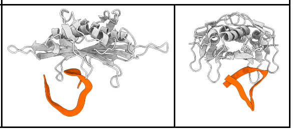 |

**Table S3.** CP-anchored RNA stem-loops. They have cross-correlation scores smaller than 0.30 to the density of the operator. Each stem-loop-CP-dimer complex is shown in two orthogonal views (left and right, respectively). The residue numbers for each stem-loop are indicated in the parenthesis.

| 2 (106-118) | 6 (436-449) | 11 (893-905) | 13 (998-1101) |
|---|---|---|---|
| 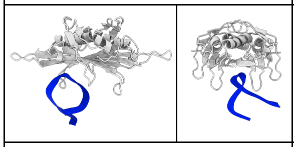 | 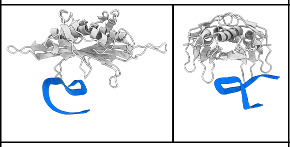 | 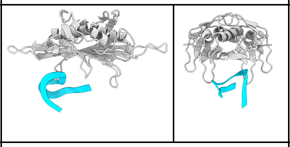 | 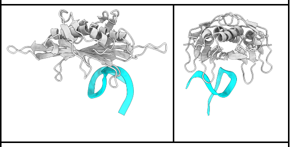 |
| 18 (1418-1431) | 19 (1447-1460) | 25 (1788-1800) | 28 (2706-2088) |
| 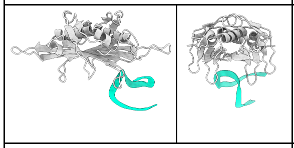 | 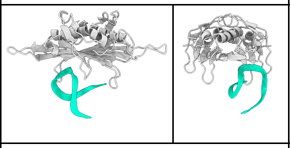 | 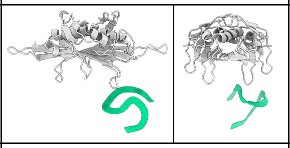 | 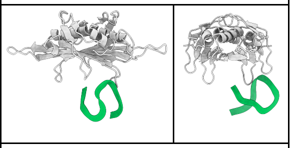 |
| 47 (3429-3442) | 48 (3485-3498) | 51 (3712-3724) | 53 (3815-3828) |
| 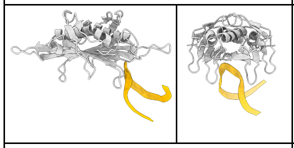 | 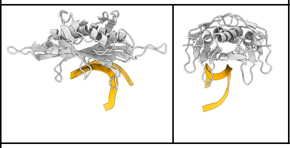 | 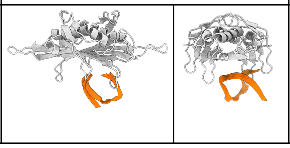 | 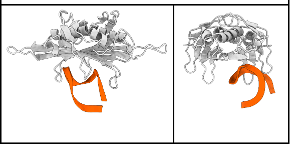 |
| 54 (3873-3886) | | | |
| 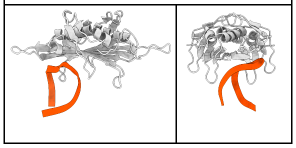 | | | |

**Table S5.** Percentages of different Qβ VLPs via different purification methods.

| | **T = 3** | **T = 4** | **Prolate** | **Oblate** | **Small Prolate** |
|---|---|---|---|---|---|
| **Wild Type (CsCl gradient)** | 97.23% | 0.45% | 1.72% | 0.60% | < 0.01% |
| **Wild Type (gel-filtration)** | 74.67% | < 0.01% | 0.97% | 21.09% | 3.27% |
| ***cp/A₁* (gel-filtration)** | 52.85% | < 0.01% | 0.29% | 35.28% | 11.58% |

**Video Legends**

**Video s1. The model of the entire Qβ virion.** The gRNA is shown as ribbons and rainbow-colored from the 5′ end (blue) to the 3′ end (red). The CP shell and Mat are represented as densities and colored light grey and dark blue, respectively.

**Video S2. The domain-by-domain fold of the Qβ gRNA.** The gRNA fragments corresponding to the 5′ UTR, mat, cp, read-through, replicase, and 3′ UTR, are colored blue, green, yellow, purple, orange, and red, respectively. The density of the gRNA is colored grey and transparent to show the fitting of the RNA structure of each gene.

**Video S3. The morphing of the conformations of hexamers from different Qβ VLPs.** The hexamers are aligned by subunit a, which is defined in **Figure 4B**.

**SI References**

1. Witherell, G. W.; Uhlenbeck, O. C., Specific RNA binding by Q beta coat protein. Biochemistry 1989, 28, (1), 71-6.
